# Prevalence of Iron-deficiency Anaemia among University Students in Noakhali Region, Bangladesh

**Published:** 2014-03

**Authors:** Kumar B. Shill, Palash Karmakar, Md. G. Kibria, Abhijit Das, Mohammad A. Rahman, Mohammad S. Hossain, Mohammad M. Sattar

**Affiliations:** ^1^Noakhali Science and Technology University, Sonapur, Noakhali, Bangladesh;; ^2^Professor, Department of Pharmacy, Jahangirnagar University, Savar, Dhaka, Bangladesh

**Keywords:** Iron-deficiency anaemia, Prevalence, University students, Bangladesh

## Abstract

Iron-deficiency anaemia (IDA) is a common health problem in rural women and young children of Bangladesh. The university students usually take food from residential halls, and the food value of their diets is not always balanced. This cross-sectional study was conducted to estimate the prevalence of iron-deficiency anaemia among the university students of Noakhali region, Bangladesh. Haemoglobin level of 300 randomly-selected students was measured calorimetrically, using Sahli's haemoglobinometer during October to December 2011. Statistical analysis was done by using SPSS software for Windows (version 16) (SPSS Inc., Chicago, IL, USA). In the study, 55.3% students were found anaemic, of whom 36.7% were male, and 63.3% were female. Students aged 20-22 years were more anaemic (43.4%) than other age-groups. Majority (51.3%) of male students showed their haemoglobin level in the range of 13-15 g/dL, followed by 26.0% and 21.3% with 10-12 g/dL and 16-18 g/dL respectively. Although 50.5% anaemic and 51.1% non-anaemic female students showed normal BMI—lower percentage than anaemic (60.7%) and non-anaemic (71.9%) male students, the underweight students were found more anaemic than the overweight and obese subjects. Regular breakfast-taking habit showed significant (p=0.035, 95% CI 0.5-1.0) influence on IDA compared to non-regular breakfast takers. Consumption of meat, fish, poultry, eggs, or peanut butter regularly; junk food; multivitamins; and iron/iron-rich food showed insignificant (p=0.097, 95% CI 0.5-1.1; p=0.053, 95% CI 1.1-2.3; p=0.148, 95% CI 0.6-1.2; and p=0.487, 95% CI 0.7-1.4 respectively) role in provoking IDA. In the case of non-anaemic subjects, all of the above parameters were significant, except the junk food consumption (p=0.342, 95% CI 0.5-1.2). Our study revealed that majority of university students, especially female, were anaemic that might be aggravated by food habit and lack of awareness. The results suggest that anaemia can be prevented by providing proper knowledge on the healthful diet, improved lifestyle, and harmful effect of anaemia to the students.

## INTRODUCTION

Anaemia can be defined as a condition in which there is a low level of haemoglobin due to either few numbers of red blood cells and/or little haemoglobin in each cell (1). Anaemia is one of the most important global health problems, and more than two billion people worldwide are estimated to have anaemia, with majority coming from the developing countries (2-4). Its adverse health consequences affect people of all age-groups and can result from non-nutritional and nutritional factors (5). It is categorized as one of the 10 most serious health problems by the World Health Organization (6).

Iron-deficiency anaemia is the most common type of nutritional anaemia which results from long-term negative iron balance and is responsible for approximately 50% of all anaemia (7-8). It is a severe stage of iron shortage in which haemoglobin (or haematocrit) falls below the normal range. It is more widespread and severe in young children and women of reproductive age but it can be found in people of any age-group. Deficiency of iron usually develops slowly and is not clinically evident until anaemia becomes severe (9). Accelerated development, hormonal changes, malnutrition, and starting of menstrual periods in girls are the major causes of iron-deficiency anaemia during adolescence, which may also lead to impaired perception and learning difficulties (10).

In Bangladesh, nutritional anaemia has long been identified as a serious public-health problem (11). The national vitamin A survey conducted in 1997-1998 showed that nearly 50% of pregnant women in rural Bangladesh had anaemia (12). Moreover, many surveys conducted in the past stated that anaemia is a severe problem among all across age, population and geographic groups in Bangladesh. In 2004, another survey conducted by Nutritional Surveillance Project of Helen Keller International in collaboration with the Institute of Public Health Nutrition showed that 68% of under-five children were anaemic. The survey also suggested that 40% of adolescent girls and 31% adolescent boys as well as 46% of non-pregnant and 39% of pregnant women were affected by anaemia (6). The anaemia prevention and control strategies have focused on correcting this deficiency by routine iron supplementation. However, the effectiveness of iron supplementation programmes has generally been low (13). All these studies have been carried out among the rural populations, and inadequate attention has been paid among the educated urban populations. The present study aimed to assess the prevalence and causes of iron-deficiency anaemia through laboratory evaluation of individual's haemoglobin level and assessment of their health condition and lifestyle through a structured questionnaire administered among the university students of Noakhali Science and Technology University, Bangladesh.

## MATERIALS AND METHODS

### Study design

The study was done using a cross-sectional design to determine the prevalence of anaemia among the university students by analyzing blood samples to measure the concentration of haemoglobin (Hb) and to evaluate their body mass index (BMI), dietary habits, and the status of awareness regarding their food intake. It was a project work for the partial fulfillment of the requirement for the degree of Bachelor of Pharmacy and was conducted during October to December 2011.

Study subjects

The study included 300 graduation-level students aged 17-25 years (150 male and 150 female), with different socioeconomic backgrounds, from 7 departments. We conducted this study with equal number of male and female students because we wanted to compare the prevalence of anaemia between these two groups. Both residential and non-residential students were selected randomly, i.e. we did not consider whether the students were resident or non-resident in different halls of the university. Before collecting blood, participants were informed about the experiment, and a verbal consent was taken from each participant. Participant who was not interested to provide his/her blood voluntarily were excluded from the study, and another volunteer was selected.

### Data collection

A team comprising one doctor, two laboratory technicians, and volunteers from the Department of Pharmacy collected data, using a pretested questionnaire and measured haemoglobin concentration, height, and weight of the individuals. The questionnaire was developed to obtain information about age, sex, and food habits. Intake of regular breakfast as well as taking meat, fish, poultry, eggs, or peanut butter regularly and frequency of the intake of junk food, multivitamins and iron/iron-rich food were included as food habits of the participants.

Haemoglobin concentrations were analyzed calorimetrically, using Sahli's haemoglobinometer. Blood samples were drawn by Sahli's pipette and then added to the haemoglobin tube where haemoglobin (Hb) was converted to acid haematin by the addition of 0.1 N hydrochloric acid, and the resulting brown colour was diluted by adding distilled water, which was finally compared with the standard brown glass reference blocks of Sahli's haemoglobinometer (14).

Body mass index (BMI) was calculated as weight in kg divided by the square of height in metre for every individual (15). Body-weight and height were measured by portable balance and measuring tape.

### Ethical consideration

This study was approved by the Department of Pharmacy and supervised by the residential doctor of Noakhali Science and Technology University. Blood samples were collected only from those students who were interested to donate their blood voluntarily. The information of each student under the study was preserved confidentially.

**Table 1.** Prevalence of iron-deficiency anaemia, with physical parameters of the study subjects

Parameter	Category	Anaemic			Non-anaemic		
		Total	%	95% CI	Total	%	95% CI
All students (N=300)		166	55.3	53.2-60.8	134	44.7	43.0-50.2
Age (completed years)							
17-19		46	27.7	27.2-28.3	16	11.9	10.9-13.0
20-22		72	43.4	43.0-43.7	65	48.5	48.2-48.8
23-25		48	28.9	28.8-29.0	53	39.6	39.2-39.9
Sex							
Male		61	36.7	36.4-37.1	89	66.4	66.2-66.6
Female		105	63.3	63.0-63.5	45	33.6	33.3-33.8
Body mass index							
Female (n=150)							
<18.5	Underweight	33	31.4	29.6-33.3	09	20.0	19.2-20.8
≥18.5 to ≤24.9	Normal	53	50.5	49.0-52.0	23	51.1	49.2-53.0
≥25 to <30	Overweight	19	18.1	17.0-19.1	13	28.9	28.4-29.4
≥30	Obese	0	0	0	0	0	0
Male (n=150)							
<18.5	Underweight	14	19.7	18.1-21.2	0	0	0
≥18.5 to ≤24.9	Normal	29	60.7	59.5-61.9	56	71.9	70.0-73.8
≥25 to <30	Overweight	12	13.1	12.2-14.0	23	18.0	16.7-19.2
≥30	Obese	06	6.5	6.2-7.0	10	10.1	9.4-10.8

CI=Confidence interval;

p<0.05 was considered significant

**Figure.**
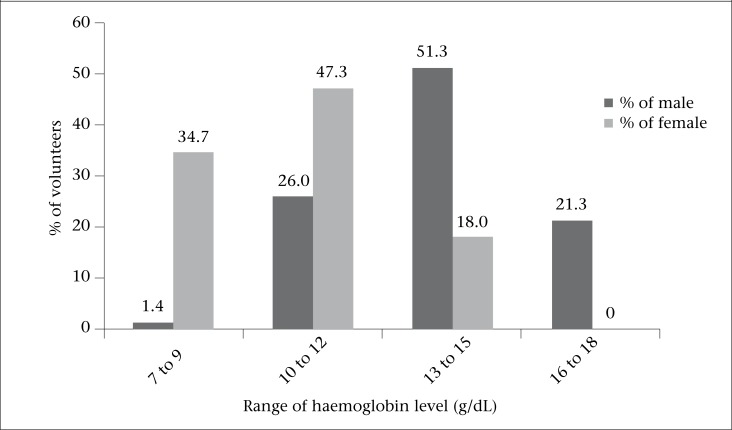
Percentage of haemoglobin concentrations between male and female students

### Statistical methods

Data-entry and analysis were done by using SPSS for Windows (version 16) (SPSS Inc., Chicago, IL, USA). Proportions and 95% confidence intervals (95% CI) were calculated using descriptive statistics. Differences between proportions were considered statistically significant if 95% CI did not overlap. The odds ratios and p values were calculated by risk estimate and chi-square test, using analytic statistics. An alpha level of 0.05 or less was considered significant.

## RESULTS

### Prevalence of anaemia

Table 1 shows the prevalence of anaemia by age, sex, and body mass index (BMI); 55.3% students were found anaemic, of whom 36.7% were male, and 63.3% were female. In the case of non-anaemic students, the percentage of male was approximately double compared to the female, i.e. 66.4% and 33.6% respectively. The prevalence of anaemia in students of 20-22 years age-group was higher (43.4%) than the 17-19 years (27.7%) and 23-25 (28.9%) years age-groups. Similarly, in the case of non-anaemic students, age-group of 20-22 years was also the higher (48.5%) than other two age-groups (17-19 years: 11.9% and 23-25 years: 39.6%). When considering BMI among the female anaemic students, about half (50.5%) were found normal-weight students, 31.4% were underweight, and 18.1% were overweight whereas, in the case of non-anaemic females, 51.1% were of normal weight, 20.0% were underweight, and 28.9% were overweight. No obese female students were found either in anaemic or non-anaemic group. On the other hand, 60.7% male anaemic students had normal body-weight, 19.7%, 13.1%, and 6.5% were underweight, overweight, and obese respectively. However, in contrast to the non-anaemic males, 71.9 % students showed normal body-weight while 18.0% and 10.1% were overweight and obese respectively; no students were found underweight.

The figure illustrates that, out of 150 male, the majority (51.3%) showed their haemoglobin levels in the range of 13-15 g/dL, 26.0% showed 10-12 g/dL, and 21.3% were found in the range of 16-18 g/dL. However, in the case of female, haemoglobin range of 10-12 g/dL was found to be the highest (47.3%), followed by 7-9 g/dL (34.7%) and 13-15 g/dL (18.0%). No female students were found with haemoglobin level of 16-18 g/dL.

Table 2 shows the prevalence of anaemia by dietary habits of the individuals. Anaemia was significantly (p= 0.035, 95% CI 0.5-1.0) more prevalent among those who were irregular in their breakfast intake than the individuals taking regular breakfast, and it was more statistically significant (p=0.0002, 95% CI 1.4-3.4) for the non-anaemic students. The number of those who were not regular eaters of certain food items, such as meat, fish, poultry, eggs, or peanut butter, was not statistically significant (p=0.097, 95% CI 0.5-1.1) for provoking anaemia but significant (p=0.01, 95% CI 1.1-2.6) for the subjects who were non-anaemic. The number of those who were taking junk food had no statistically significant (p=0.053, 95% CI 1.1-2.3) relationship with developing anaemia. Multvitamins consumption was not statistically significant (p=0.148, 95% CI 0.6-1.2) for anaemic subjects but significant (p=0.041, 95% CI 0.4-1.0) for non-anaemic subjects. On the basis of iron/iron-rich food consumption frequency (regularly and irregularly), there was a very little difference among the anaemic subjects, which was not significant (p=0.487, 95% CI 0.7-1.4) but a significant (p<0.0001, 95% CI 1.9-4.6) difference was observed among the non-anaemic students in terms of regular and irregular intake of iron/iron-rich food.

Table 3 presents the awareness of anaemia among the university students. Among the male students, significantly higher proportion [74.0% (111/150); p<0.0001, 95% CI 0.2-0.5)] of students were unaware whereas 26.0% (39/150) were aware about anaemia. In the case of female students, the difference in awareness (45.3%, 68/150) and unawareness (54.7%, 82/150) was not statistically significant (p=0.369, 95% CI 0.6-1.2).

## DISCUSSION

Anaemia, especially iron-deficiency anaemia, is the most common nutritional deficiency worldwide. The detrimental effects of anaemia on work productivity of adults and physical development of children are of major concern (16).

In this study, 55.3% students were anaemic, of whom 63.3% were female; thus, anaemia was found to be much more common among females than males (36.7%, p<0.0001). Possible reasons might be poor dietary habit, menstrual blood loss, and lack of awareness of iron-deficiency and nutritional status (1). Several studies showed that, as in Bangladesh, university students of other countries were also affected by iron-deficiency or iron-deficiency anaemia. In Indian medical students, prevalence of IDA was found 32.0%, of whom 44.0% were girls, and 20.0% were boys. Besides, the prevalence of iron-deficiency was 40.9%, and that of IDA was 3.8% in Iranian university students; anaemia prevalence rate was also found to be 29.0% in Emirati students of 18-24 years age-group (17-19). Females are prone to anaemia because of menstruation and due to social customs; they get a diet of inferior quality compared to males. With the substantial differences between the sexes, total body iron stores contain about 2 to 4 g and lose 1 mg of iron per day (20). However, 42 mg of iron loss per menstrual cycle has been reported in females with heavy blood flow, which may lead to anaemia (21). On the other hand, males suffer from anaemia mostly due to poor diet. About half of both anaemic and non-anaemic students showed normal body-weight. Overweight females were less anaemic than the underweight ones. Similarly, among the male anaemic students, the underweight individuals were more prevalent than overweight and the obese, which suggests that overweight and obese people are less likely to be anaemic compared to normal and underweight people (22).

**Table 2.** Prevalence of iron-deficiency anaemia by dietary habit among the study subjects

Parameter	Anaemic (n=166)				Non-anaemic (n=134)			
	Total	%	p value	Odds ratio (95% CI)	Total	%	p value	Odds ratio (95% CI)
Regular breakfast intake								
Yes	68	41.0	0.035	0.7 (0.5-1.0)	92	68.7	0.0002	2.2 (1.4-3.4)
No	98	59.0			42	31.3		
Eat meat, fish, poultry, eggs, or peanut butter regularly								
Yes	72	43.4	0.097	0.8 (0.5-1.1)	84	62.7	0.01	1.7 (1.1-2.6)
No	94	56.6			50	37.3		
Eat junk food								
Yes	102	61.5	0.053	1.6 (1.1-2.3)	60	44.8	0.342	0.8 (0.5-1.2)
No	64	38.5			74	55.2		
Take multivitamins								
Yes	74	44.6	0.148	0.8 (0.6-1.2)	54	40.3	0.041	0.7 (0.4-1.0)
No	90	55.4			80	59.7		
Intake frequency of iron/iron-rich food								
Regularly	82	49.4	0.487	1.0 (0.7-1.4)	100	74.6	<0.0001	2.9 (1.9-4.6)
Irregularly	84	50.6			34	25.4		

CI=Confidence interval;

p<0.05 was considered significant

**Table 3.** Awareness of iron-deficiency anaemia between male and female students

Awareness level	Male (n=150)				Female (n=150)			
	No. of res-pondents	%	p value	Odds ratio 95% CI	No. of res-pondents	%	p value	Odds ratio 95% CI
Aware	39	26.0		0.4	68	45.3		0.8
			<0.0001				0.369	
Unaware	111	74.0		(0.2-0.5)	82	54.7		(0.6-1.2)

CI=Confidence interval;

p< 0.05 was considered significant

In our study, regular breakfast intake revealed statistically significant difference among anaemic (p=0.035, 95% CI 0.5-1.0) and non-anaemic (p=0.0002, 95% CI 1.4-3.4) students. Among the anaemic students 41.0% were regular in their breakfast intake and, in the case of non-anaemic students, the figure was 68.7%. A nutritious breakfast that includes sugar, starch, protein, fat, fibre, vitamins and minerals, especially iron and vitamin C, is necessary to ensure the sustained release of energy. Skipping breakfast is a known practice among university students of Bangladesh due to late awaking, not being hungry in the morning, or disliking towards the food served. This not only exists in Bangladesh but also found in the Gulf countries (23-24).

The regular (43.4%) and irregular (56.6%) consumption of meat, fish, poultry, eggs, or peanut butter was not statistically significant (p=0.097, 95% CI 0.5-1.1) among the anaemic students but it was significant (p=0.01, 95% CI 1.1-2.6) among the non-anaemic subjects, in which case, the rates of regular and irregular intake of the abovementioned diet were 62.7% and 37.3% respectively. Another study revealed similar results that low consumption of diets, such as red meat, vegetables, fruits, and cereals has been reported to be associated with iron-deficiency anaemia (25). The present study showed that junk food consumption among the anaemic and the non-anaemic subjects had no statistically significant influence on IDA (p=0.053, 95% CI 11-2.3 and p=0.342, 95% CI 0.5-1.2 respectively). Out of the anaemic subjects, 61.5% preferred junk food while it was 44.8% for the non-anaemic subjects. The traditional diet of Bangladesh has changed with the introduction of junk food, and numerous studies have attributed iron-deficiency anaemia to the change in dietary habits (26).

The present study showed that the consumption of multivitamins was not statistically significant (p=0.148, 95% CI 0.6-1.2) in the case of anaemic subjects and was significant (p=0.041, 95% CI 0.4-1.0) for non-anaemic subjects. Another study showed that multivitamin-mineral supplementation is not very efficacious in improving haemoglobin concentration (27).

Iron/iron-rich food intake frequency was statistically insignificant for the anaemic subjects (p=0.487, 95% CI 0.7-1.4) while it was significant in case of non-anaemic subjects (p<0.0001, 95% CI 1.9-4.6). Among the anaemic subjects, 49.4% consume iron/iron-rich food regularly, and 50.6% were irregular but, in the case of non-anaemic subjects, 74.6% were regular, and 25.4% were irregular. Iron deficiency is the major public-health concern in the developing countries. The size and number of red blood cells are reduced and cause iron-deficiency anaemia which affects the function of several organic systems. So, proper supplementation of iron is needed to reduce the prevalence of iron-deficiency anaemia. In recent years, a number of studies have suggested that, in various groups at risk of iron-deficiency anemia, weekly iron supplementation was as effective as daily iron supplementation in raising the haemoglobin levels (28-29).

Findings of other studies indicated that, although all of the subjects were educated, about half of them were anaemic. Results of this study reflect the scenario of poor dietary habit and nutritional awareness responsible for IDA among students of a university in Bangladesh. As residential dietary facilities in most universities in Bangladesh are almost the same, it can be mentioned that students of other universities might also be affected by IDA. According to our findings, only proper nutritious food and awareness can prevent IDA. The findings may be helpful in conducting more such research among the students of other universities to estimate the prevalence of IDA as well as improving the awareness of taking balanced diets, benefits of nutritious food, especially iron-rich food, and healthy lifestyle to prevent IDA.

### Limitations

The possible limitation of this study was that, due to lack of fund and more resource personnel, we included only the students of Noakhali Science and Technology University in the study for collecting data, although some other colleges were present in this region. Another possible limitation was the use of Sahli's haemoglobinometer to measure haemoglobin concentration calorimetrically whereas HemoCue or other sophisticated device could be used for getting more accurate results of haemoglobin concentration. With haemoglobin, other haematological parameters, such as haematocrit, serum ferritin, mean corpuscular haemoglobin concentration, red blood cell count, etc. were not measured due to the lack of facilities and resources. Funding is essential to conduct this type of research not only in this region but also in any other parts of the country to get more specific and versatile results.

### Conclusions

Iron-deficiency anaemia is predominant among a large number of people, especially rural women and children in Bangladesh. In most of the cases, it occurs due to the lack of iron-rich food in daily diet and, sometimes, excess menstrual blood loss for women. The present study indicates that, besides the rural women, the majority of university students, especially female, are affected by iron-deficiency anaemia. The possible reason might be their food habit which provides insufficient amount of iron. Irregular intake of breakfast and iron supplement may be another reason behind this. Although the study subjects are university students, it is unfortunate that majority of them are not conscious about anaemia. Proper health education to increase knowledge about anaemia as well as its causative factors, detrimental effect of clinical outcomes, benefits of taking iron-rich food, and avoiding unhealthy food, including fast/junk food, will be helpful to the students. The Government of Bangladesh should take initiatives to assess the prevalence of iron-deficiency anaemia among the students of public and private universities. Providing iron-rich and nutritious foods for all residential students in their daily diets will be beneficial to prevent anaemia.

## ACKNOWLEDGEMENTS

The authors are thankful to the Department of Pharmacy, Noakhali Science and Technology University, for providing the laboratory facilities for doing the research work. Authors also would like to thank all the volunteer students, laboratory staff of the Department of Pharmacy, and staff of the medical centre for their cordial support to this research work.
